# Leading the World’s Premier Environmental Health Organization: A Message from Linda Birnbaum

**DOI:** 10.1289/ehp.12670

**Published:** 2009-04

**Authors:** Linda S. Birnbaum

**Affiliations:** National Institutes of Health, Department of Health and Human Services, Research Triangle Park, North Carolina, E-mail: birnbaumls@niehs.nih.gov

I am thrilled to be back at the National Institute of Environmental Health Sciences (NIEHS). I have always viewed NIEHS as the world’s premier environmental health research organization. Although it has been 19 years since I daily roamed the NIEHS halls and worked side by side with many of the scientists here, I still feel that NIEHS pride. I am especially pleased to be serving as the director of both the NIEHS and the National Toxicology Program (NTP) during a national movement for positive, constructive change in our government and in our country, and at a time when health and the environment are top priorities.

I know everyone is wondering what the institute’s research priorities will be under my leadership and what kind of management style I will use to achieve our shared vision. As director, I’m not planning any major changes right away. I want to take some time and learn as much as I can from as many voices as I can to determine the true needs of the institute and to better understand the communities we serve. Once I am armed with information and knowledge, I will move forward in making decisions to improve the institute’s operation and expand the effectiveness of our research. I plan to focus my full attention on running the institute and will not establish a laboratory anytime in the near future. As someone who values open communication and transparency, I will work on restoring trust in the leadership team and focus on building a more cohesive NIEHS. I am interested in empowering those who work for and with me, and building a team that will share my vision and challenge me. We will conduct broad national searches to fill all of the key open positions at the institute. My first priority will be to make NIEHS even better than it already is.

Another priority crucial to NIEHS’s success will be to establish or reestablish links with others involved in environmental health research efforts. We will be working to cross-fertilize our own science teams at the institute, for example, by creating more synergy and research opportunities among intramural scientists, NTP scientists, and extramural grantees. We will work to restore and foster new relationships with other NIH institutes, federal agencies (e.g., Centers for Disease Control and Prevention, U.S. Environmental Protection Agency, Food and Drug Administration, Department of Energy), universities, community and advocacy groups, professional societies, members of Congress, the media, other stakeholders, and, of course, those who pay our bills—the general public. Realizing that communication is a two-way street, we will look for ways to create more opportunities to share our science findings with our constituents and, in turn, to listen to what our constituents want us to hear. We will invite and welcome as many stakeholders and collaborators as we can to our home base in Research Triangle Park, North Carolina, and we plan on coming to you, to your meetings, your facilities, your websites, blogs, and podcasts, your virtual worlds, your laboratories, and any other venues we can visit. We will continue our outreach and support efforts for grantees and other constituencies, and we will be looking for ways to make America’s tax dollars go even further, for example, by sharing resources with neighboring agencies and facilities.

I see environmental health as a global issue that needs to be tackled from many angles. Understanding how the environment impacts health, and then using that knowledge to improve health worldwide, is not something we can achieve on our own. We need to make the whole bigger than the sum of its parts, meaning we need individuals and we need teams to integrate across disciplines and work together to translate basic science into improved health for all. For example, by looking at multiple disease inputs and understanding how a certain environmental chemical or stressor can cause a disease process, researchers can prevent or stop the progression of many complex health problems. We will also tackle environmental issues by continuing to move our programs and tools into the 21st century and by finding ways to respond more rapidly and efficiently to address emerging health threats.

As for *Environmental Health Perspectives* (*EHP*), I have always been a strong supporter of the journal and feel extremely fortunate to have it as part of the NIEHS portfolio. As a scientist, I have had more than two dozen articles go through the *EHP* peer review process and be published. I value that *EHP* is read by such a diverse audience, from bench scientists, toxicologists, and chemists to epidemiologists, clinicians, economists, and students all over the world. With the highest impact factor of any environmental journal, it keeps readers abreast of the latest science and is reflective of the latest trends in science.

I am excited and proud to be the director of NIEHS and NTP, and I’m looking forward to finding creative new ways to foster our two- way communication and make NIEHS the best research institution it can be.

## Figures and Tables

**Figure:**
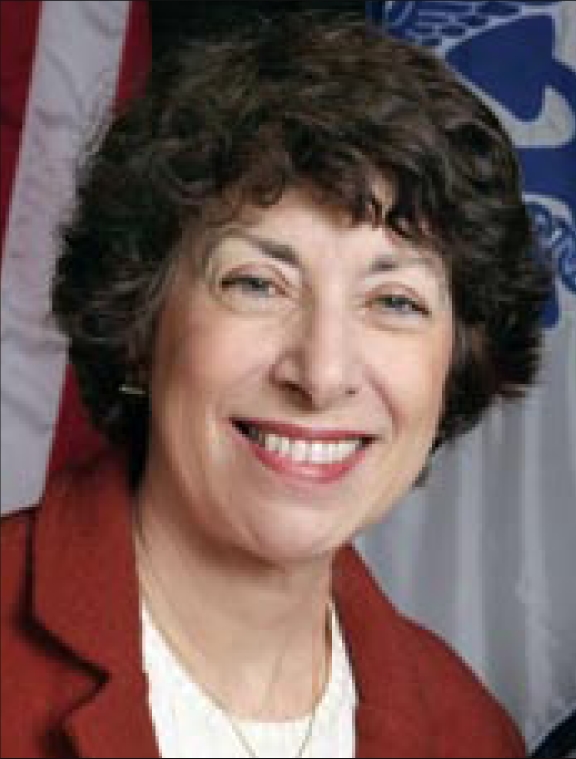
Linda S. Birnbaum

